# Automated Pulmonary Nodule Classification in Computed Tomography Images Using a Deep Convolutional Neural Network Trained by Generative Adversarial Networks

**DOI:** 10.1155/2019/6051939

**Published:** 2019-01-02

**Authors:** Yuya Onishi, Atsushi Teramoto, Masakazu Tsujimoto, Tetsuya Tsukamoto, Kuniaki Saito, Hiroshi Toyama, Kazuyoshi Imaizumi, Hiroshi Fujita

**Affiliations:** ^1^Graduate School of Health Sciences, Fujita Health University, 1–98 Dengakugakubo, Kutsukake-cho, Toyoake City, Aichi 470-1192, Japan; ^2^Fujita Health University Hospital, 1–98 Dengakugakubo, Kutsukake-cho, Toyoake City, Aichi 470-1192, Japan; ^3^School of Medicine, Fujita Health University, 1–98 Dengakugakubo, Kutsukake-cho, Toyoake City, Aichi 470-1192, Japan; ^4^Gifu University, 1–1 Yanagido, Gifu 501-1194, Japan

## Abstract

Lung cancer is a leading cause of death worldwide. Although computed tomography (CT) examinations are frequently used for lung cancer diagnosis, it can be difficult to distinguish between benign and malignant pulmonary nodules on the basis of CT images alone. Therefore, a bronchoscopic biopsy may be conducted if malignancy is suspected following CT examinations. However, biopsies are highly invasive, and patients with benign nodules may undergo many unnecessary biopsies. To prevent this, an imaging diagnosis with high classification accuracy is essential. In this study, we investigate the automated classification of pulmonary nodules in CT images using a deep convolutional neural network (DCNN). We use generative adversarial networks (GANs) to generate additional images when only small amounts of data are available, which is a common problem in medical research, and evaluate whether the classification accuracy is improved by generating a large amount of new pulmonary nodule images using the GAN. Using the proposed method, CT images of 60 cases with confirmed pathological diagnosis by biopsy are analyzed. The benign nodules assessed in this study are difficult for radiologists to differentiate because they cannot be rejected as being malignant. A volume of interest centered on the pulmonary nodule is extracted from the CT images, and further images are created using axial sections and augmented data. The DCNN is trained using nodule images generated by the GAN and then fine-tuned using the actual nodule images to allow the DCNN to distinguish between benign and malignant nodules. This pretraining and fine-tuning process makes it possible to distinguish 66.7% of benign nodules and 93.9% of malignant nodules. These results indicate that the proposed method improves the classification accuracy by approximately 20% in comparison with training using only the original images.

## 1. Introduction

### 1.1. Background

Lung cancer is a leading cause of death worldwide [1]. The disease exhibits a rapidly worsening prognosis with progression, necessitating early detection and treatment. Chest radiography and sputum cytology are widely used screening techniques for the early detection of lung cancer. If the screening examination reveals suspicious findings, computed tomography (CT) is performed to provide a more detailed assessment [2]. CT examinations have high detection ability and can even reveal small lesions; therefore, they are indispensable for lung cancer diagnosis [3]. However, it is often difficult to distinguish between benign and malignant pulmonary nodules on the basis of CT images alone. Therefore, a bronchoscopic biopsy is conducted if the CT examinations indicate malignancy. However, bronchoscopic biopsies are highly invasive and are associated with the risk of complications and infectious diseases, because tissues and cells are collected directly from the patient's body [4]. This approach also entails multiple unnecessary biopsies for patients with benign nodules. Thus, an imaging diagnosis with high classification accuracy is essential.

Computer-aided diagnosis (CAD) is a useful tool for reducing the incidence of biopsies, as it supports diagnosis by allowing quantitative analysis of medical images with a computer.

In studies on the use of CAD in lung cancer diagnosis, various methods have been developed for calculating hand-crafted features such as nodule shape, and machine learning has been used to distinguish between benign and malignant nodules [5–7]. Hand-crafted feature extraction may focus on the characteristic shapes of nodules. Thus, cases that do not correspond to the characteristics may be difficult to classify, and the addition of new features to improve accuracy may also be difficult. Many recent studies have proposed classification methods based on deep convolutional neural network (DCNN), which is a deep learning network [8] that offer excellent image recognition ability [9–12]. Satisfactory results have been obtained by automating the conventional feature extraction approach, even for difficult cases. A recent trend involves training a DCNN using a deep layer such as ResNet [13] and DenseNet [14], which are frequently used in the field of natural images. A deeper neural network may increase the power of expression [15]. However, the training necessary to obtain a deeper neural network requires a sufficient amount of training data, and the amount of available medical imaging data is limited by ethical issues and time constraints. Thus, the limited availability of medical imaging data in comparison with the datasets used for other image classification tasks presents a problem.

To overcome this problem, we focus on generative adversarial networks (GANs) [16]. A GAN is an artificial intelligence model that generates new images that are similar to training images. The GAN has two networks, called the generator and discriminator; the generator tries to generate images similar to the training data, whereas the discriminator tries to classify whether the generated image is real or fake. The discriminator's network is backpropagated so that errors can be reduced and classification accuracy can be improved. In contrast, the generator's network is optimized to incorrectly classify the generated images. Thus, GAN training is performed by two competing networks. Using this technique, enormous volumes of data can be generated from medical images with limited amounts of original data. The generated images can also be applied to DCNN training. It is expected that this approach will avoid ethical problems, as the generated images do not correspond to real patients.

### 1.2. Studies Related to GAN

GANs have recently been applied in various fields, and many studies are also being conducted on medical images. Most studies on GANs have employed image-to-image translation [17–19]. For example, Costa et al. [17] performed vessel segmentation using U-Net and generated new retinal images using GANs. In addition, good results have been reported in cross-modality translations, such as translation from CT images to PET images [18] and translation from MRI images for treatment planning to CT images [19]. Although a fully convolutional network (FCN) is generally used in research on segmentation technology, methods using GANs have also been developed in this context, such as that by Dai et al. [20], who performed the segmentation of lung and heart areas on chest X-ray images using GANs. GANs have also been used to reduce the noise in CT images, for example, by Yang et al. [21], who effectively improved the problem of oversmoothing by reducing noise from low-dose CT images. Thus, processes such as segmentation and noise reduction, which were conventionally performed by various methods, have been successfully improved by using GANs.

However, few studies have assessed the use of GANs to improve limited amount of data, which is the problem we aim to solve here [22, 23]. Frid-Adar et al. [22] increased the training data available for liver lesion classification using a GAN with a DCNN. Salehinejad et al. [23] also increased the number of training samples by using a GAN for lesion classification in chest X-ray images. Furthermore, the bias between the normal and abnormal cases was balanced by changing the number of images generated by the GAN [23]. In both studies, the classification accuracy was improved by increasing the available data. In these methods, DCNN training was performed using the original images and the GAN-generated images together. Although GAN-generated images are meant to compete with the real images, some may be obviously dissimilar to real images and can be easily identified by the human eye. Moreover, some of the GAN-generated images may be blurred, having a different resolution from the real images. Therefore, the simultaneous use of real and generated images for training may be problematic. Hence, we develop a new method that employs a GAN to increase the set of image data.

### 1.3. Objective

The GAN-generated images were not used to train the DCNN alongside real images, but were instead used for pretraining. Fine-tuning of the DCNN then proceeded using the original nodule images. This stepwise training approach allows the acquisition of rough image features for lung nodules from the GAN-generated images, followed by the adjustment of finer parameters using the original nodule images. Therefore, in this study, we develop a pretraining method using GAN-generated images to improve DCNN performance in the classification of pulmonary nodules.

## 2. Materials and Methods

### 2.1. Outline

**Figure 1:**
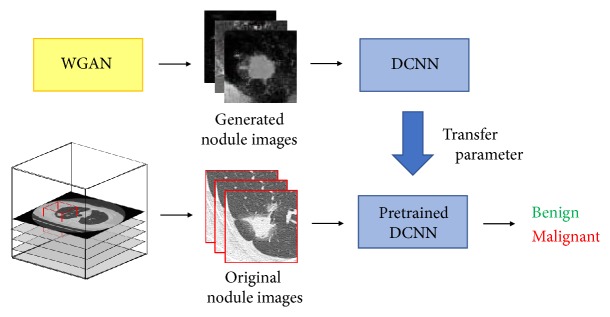
Outline of the proposed method to distinguish between benign and malignant nodules.

Figure 1 shows an outline of the proposed method. The volume of interest (VOI) centered on the pulmonary nodule is extracted from CT images, and images are created with the axial section and augmented data. To train the DCNN, pretraining is initially performed with Wasserstein GAN (WGAN)-generated nodule images. Benign and malignant nodules are then distinguished by fine-tuning using the pretrained DCNN.

### 2.2. Image Dataset

**Figure 2:**
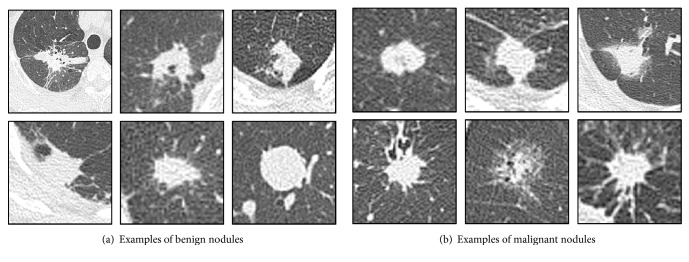
Examples of analytical cases.

The CT dataset used in this study was obtained from Fujita Health University Hospital, Japan. CT images of 60 cases with pathological diagnosis confirmed by biopsy were analyzed. They consisted of 27 cases of benign nodules and 33 cases of malignant nodules, examples of which are shown in Figure 2. The benign nodules used in this study were targeted to be difficult for radiologists to differentiate because they could not be rejected as being malignant. Aquilion ONE (Canon Medical Systems) was used as the CT scanner, and the reconstruction function was used under the condition that the lung field could be clearly observed. This study was approved by an institutional review board, and patient agreement was obtained under the condition that all data were anonymized.

First, the VOI was extracted such that the nodule was at the center. The pulmonary nodule area to be analyzed was specified by the radiologist. The VOI was cube-shaped, and the size to be extracted was twice the maximum diameter of the nodule, taking into consideration information regarding the surrounding structure of the nodule. Next, axial images were created from the center of the extracted VOI. However, with only axial images, the number of images obtained from each case is small. Training a DCNN requires sufficient training data, and small amounts of training data may result in overfitting. To prevent such overfitting, the training data were augmented by image manipulation [24, 25]. To consider information from adjacent slices, sectional images of the pulmonary nodule were created, where the slice angles varied from -40° to +40° in steps of 5° with reference to the axial images. Furthermore, by rotating and inverting the created sectional images, the training data were further augmented to serve as input images to the WGAN and DCNN.

### 2.3. Generating Pulmonary Nodules

The GAN methods previously applied to medical research often use deep convolutional GANs (DCGANs), which use a DCNN to determine the structure of the generator and discriminator [26]. In the conventional GAN method, training is performed using the Jensen–Shannon divergence to express the distance between two probability densities. This method may cause the training process to be unstable because of the vanishing gradient problem and the possibility of mode collapse with the generation of similar images. WGANs were introduced to address these issues [27]. In WGANs, the loss function is defined using the Wasserstein distance instead of the Jensen–Shannon divergence. Thus, the gradient should not disappear near the optima parameter value, which stabilizes the training process.

**Figure 3:**
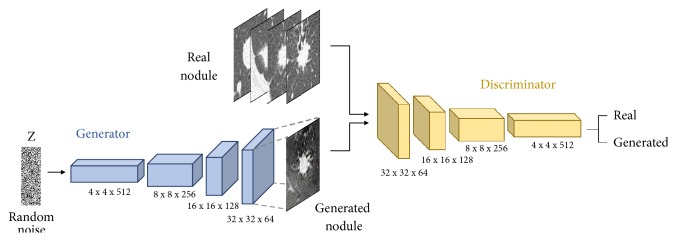
Architecture of the GAN used for nodule generation.

Figure 3 shows the architecture of the WGAN used to generate the image dataset. The generator is composed of four fractionally strided convolution layers. The generator network takes a vector of 100 random numbers drawn from a uniform distribution as input and outputs a nodule image of 64 × 64 pixels. In the fractionally strided convolution layer, the image is upsampled using zero-padding and a 5 × 5 kernel size. Batch-normalization is applied to each layer of the network, except the output layer [28, 29]. In batch-normalization, GAN training is stabilized by applying normalization throughout the minibatch. The discriminator is composed of four convolution layers; an image of 64 × 64 pixels is input to the discriminator, which determines whether the given image is real or fake. The convolution layers have a 5 × 5 kernel size, and batch-normalization is applied to each layer.

We trained the WGANs separately for benign and malignant nodules. In the real world, there exist variations such as isolated nodules and nodules infiltrating the pleura. However, if the WGANs were trained collectively for such instances, nodular images would not be generated successfully. Therefore, training was mainly carried out by selecting isolated nodules. The number of epochs and the learning rate were set to 1000 and 0.00005, respectively. RMSprop was used as the optimization algorithm. WGAN training was conducted on Ubuntu 16.04 using TensorFlow and Keras as deep learning APIs. The calculation was accelerated by a graphics processing unit (NVIDIA Quadro P5000 with 16 GB of memory).

### 2.4. Classification of Pulmonary Nodules

**Figure 4:**
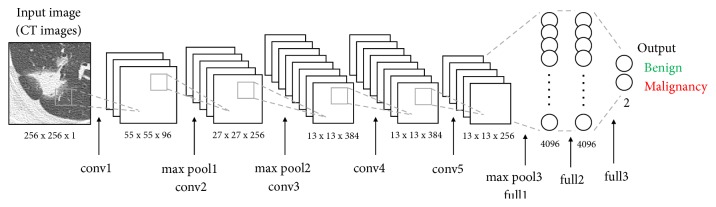
Architecture of the DCNN used for pulmonary nodule classification.

Figure 4 shows the architecture of the DCNN that was pretrained by the pulmonary nodule images generated by WGAN. This DCNN model, based on AlexNet, consists of five convolution layers, three pooling layers, and three fully connected layers. The images are resized to 256 × 256 pixels before being fed to the DCNN input layer. Convolution layer 1 uses 96 filters with an 11 × 11 kernel, resulting in a feature map of 55 × 55 × 96 pixels. Pooling layer 1 conducts subsampling that outputs the maximum value in a 3 × 3 kernel for every two pixels, reducing the matrix size of the feature map to 27 × 27 × 96 pixels. Thus, after applying five convolution layers and three pooling layers, the two-dimensional feature map input to the fully connected layer in one-dimensional form. In the three fully connected layers, we employ the dropout method (dropout rate = 0.5) to prevent overfitting. The result is a DCNN trained by a large number of nodule images generated using the WGAN.

Next, fine-tuning is conducted using the pretrained DCNN. The original pulmonary nodule images are used as the input, and the fully connected layers of the pretrained DCNN are replaced. The other parts of the DCNN are retrained using the trained parameters as initial values, with the number of epochs and learning rate set to 30 and 0.0001, respectively. Stochastic gradient descent (SGD) is used as the optimization algorithm. In this study, the DCNN was trained using the dedicated training program bundled in the Caffe package [30] on Ubuntu 16.04 and accelerated by a graphics processing unit (NVIDIA Quadro P5000 with 16 GB of memory).

## 3. Results

### 3.1. Images Generated by WGAN

**Figure 5:**
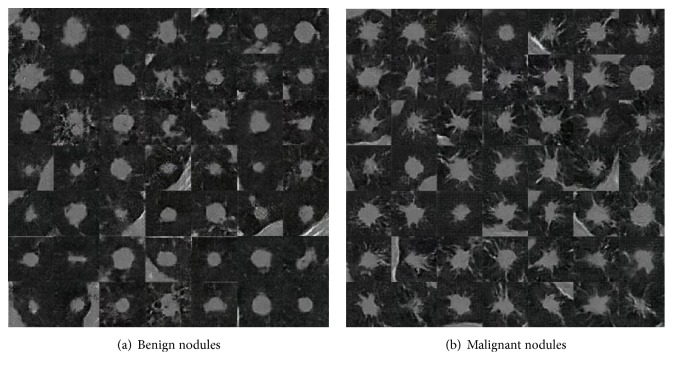
Examples of images generated using WGAN.

Examples of pulmonary nodule images generated by the WGAN are shown in Figure 5. In this study, 100,000 images of pulmonary nodules were generated. The GAN-generated images included nodule features such as spicula, and image information of the chest wall was also expressed.

### 3.2. Classification Results

For the classification of pulmonary nodules, the DCNN was trained and evaluated using the data generated by the WGAN and the original data, respectively. The classification performance was evaluated via threefold cross-validation. In this process, training images were randomly divided into three groups. The numbers of original and augmented images in each dataset used for training the WGAN and DCNN are listed in Table 1.

**Figure 6:**
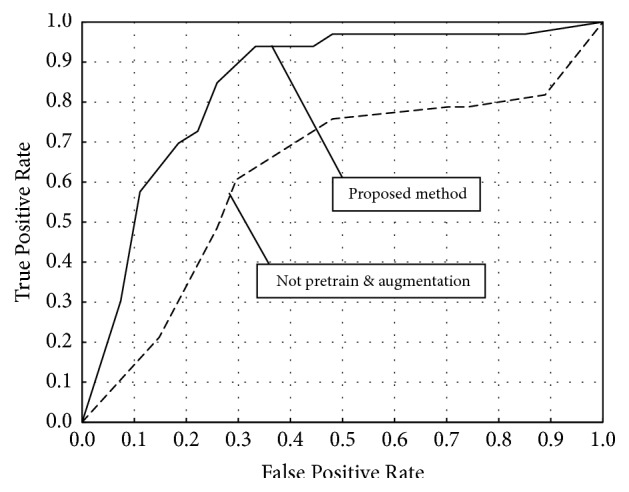
ROC curve of the proposed method.

Table 2 presents the classification results with various numbers of images used for the training stage. We constructed five datasets containing up to 100,000 images by increasing the number of pulmonary nodule images generated by WGAN. The classification accuracy is presented for each of the five sets used in pretraining.  Table 3 gives the classification results for each of the pretraining methods with and without data augmentation. We also conducted the pretraining stage using ImageNet [31], which is natural image dataset with a large amount of data, to examine the effect of pretraining using GAN-generated images. From Table 2, when the number of images generated by WGAN is 60,000, the classification accuracy of benign nodules reached 66.7% and that of malignant nodules was 93.9%, which are the highest scores in both categories. Increasing the number of generated images to 80,000 decreased the classification accuracy.  Table 3 indicates that pretraining using GAN produces better classification results than pretraining with ImageNet. In addition, data augmentation enhances the accuracy in all cases.  Figure 6 shows the receiver operating characteristic (ROC) curve drawn by changing the threshold of malignant probability from 0 to 1. The area under the curve (AUC) of the proposed method is 0.841, whereas that without pretraining and augmentation is 0.622.

Table 4 compares the performance of other network models. Among these models, the method using GAN-generated images for pretraining is the most accurate, and AlexNet produces better classification accuracy than GoogLeNet and VGG16.

**Figure 7:**
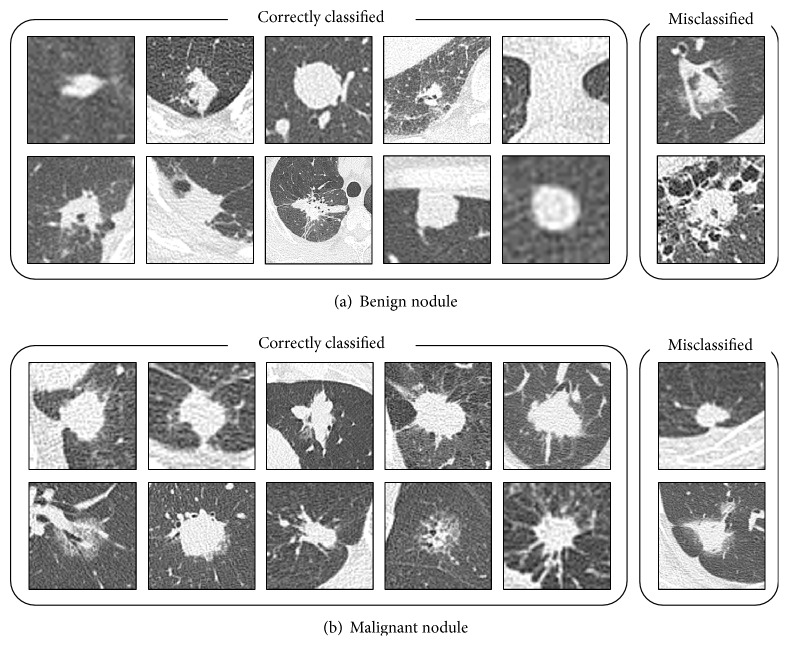
Sample images of correctly classified and misclassified nodules.

Figure 7 shows the image classification results from AlexNet when pretraining was performed using 60,000 WGAN-generated images. This approach correctly classified even the difficult nodules.

## 4. Discussion

As shown in Figure 5, the WGAN generated images that captured the benign and malignant nodule features. Benign nodules often have a rounded shape, and malignant images could also be generated with spicula and withdrawal into the pleura. In a previous study, pulmonary nodule images generated by a DCGAN succeeded in deceiving a radiologist, indicating the usefulness of the images generated by GANs [32]. In our proposed method, the WGAN generates more accurate images of pulmonary nodules, and although no visual evaluation was not conducted by a radiologist, the results are worthy of evaluation. However, unlike the original CT images, the generated images have a low resolution of 64 × 64 pixels. For natural images, GAN technology has been reported to generate images with resolutions of up to 1024 × 1024 pixels [33]. Newly improved WGAN methods have also been developed [34]. Thus, applying these methods to generate high-resolution pulmonary nodule images may lead to significant improvements in classification performance.

From Table 2, some 67% of benign nodules and 94% of malignant nodules could be distinguished using pretraining with 60,000 GAN-generated images and fine-tuning of the pretrained DCNN. In comparison with the results obtained by training with the full scratch without pretraining, the proposed approach improves the accuracy for benign nodules by 15% and that for malignant nodules by 9%. Pretraining with pulmonary nodule images generated by GAN aids the acquisition of characteristic information on benign and malignant nodules. However, when more than 60,000 images were used for pretraining, the classification accuracy dropped. We think that this is because similar nodule images are more likely to be included in the training data as the number of pretraining images increases, possibly leading to overfitting. It is necessary to investigate this problem in detail in the future. The lower identification rate for benign nodules may be because there were fewer benign cases in the original images. Therefore, we plan to increase the number of benign cases used in future studies.

As relatively few cases (60) were used in this study, training using only the original data, with no data augmentation and pretraining, yielded correct identification rates of 26% and 79% for benign and malignant nodules, respectively. Even after pretraining using ImageNet, the rates for benign and malignant nodules only improved by a few percent. However, pretraining using WGAN-generated images improved the rates for benign and malignant nodules by around 37% and 3%, respectively. Furthermore, additional data augmentation improved the rates for benign and malignant nodules by around 4% and 12%, respectively. Data augmentation and pretraining with WGAN-generated images improved the accuracy rates for benign and malignant nodules by 40.8% and 15.1%, respectively. These results suggest that the classification accuracy can be improved by GAN technology, even for medical images with limited data.

The cases used in this study could not be classified by image diagnosis alone and were eventually identified by bronchoscopic biopsy, indicating that even radiologists found them difficult to distinguish. The actual diagnostic process for lung cancer is based on a variety of patient information such as the incidence of cough and chest pain and the experience of the respiratory specialists and radiologists who examine the images. However, the DCNN in the proposed method could classify pulmonary nodules with high accuracy on the basis of CT images alone. Moreover, under actual clinical conditions, biopsies provide a definitive diagnosis but are highly invasive procedures. Thus, patients with benign nodules may undergo unnecessary biopsies. With the proposed method, almost all cases of malignant nodules and two-thirds of benign nodules were classified correctly. These results indicate that the proposed method may reduce the number of biopsies in patients with benign nodules that are difficult to differentiate from CT images by over 60%.

In this study, we used original image database. Here, CT image database is constructed in various projects such as LIDC-IDRI [35]. At the moment, the number of cases confirmed by biopsy or surgery is still small. In future, we would like to evaluate the LIDC dataset when we can use the sufficient number of cases that performed the final diagnosis. In addition, although this method involves the analysis of only the CT images, we would like to investigate whether classification accuracy can be improved further by considering various information such as positron emission tomography (PET) information, in future.

## 5. Conclusion

In this study, we developed a pretraining method using a GAN for improving the DCNN classification performance of pulmonary nodules. Experiments showed a correct identification rate of 67% for benign nodules and 94% for malignant nodules. Thus, almost all cases of malignancy were classified correctly, with two-thirds of the benign cases being also classified correctly. These results indicate that the proposed method may reduce the number of biopsies in patients with benign nodules that are difficult to differentiate from CT images by over 60%. Furthermore, the classification accuracy was clearly improved by the use of GAN technology, even for medical datasets that contain relatively few images.

## Figures and Tables

**Table 1:** Number of images in each dataset for cross-validation.

Train	Type	Set 1		Set 2		Set 3	
		Original	Augmented	Original	Augmented	Original	Augmented
WGAN	Benign	5	1280	6	1536	6	1536
	Malignant	11	2816	11	2816	11	2816

DCNN	Benign	9	2304	9	2304	9	2304
	Malignant	11	2816	11	2816	11	2816

**Table 2:** Classification results for various numbers of images used for pretraining.

The number of generated images	Classification accuracy [%]	
	Benign	Malignant
0	51.9	84.9
20,000	51.9	93.9
40,000	63.0	93.9
60,000	66.7	93.9
80,000	55.6	84.8
100,000	63.0	84.8

**Table 3:** Classification result by the pretraining method.

Pretraining method	Data augmentation	Classification accuracy [%]	
		Benign	Malignant
None	No	25.9	78.8
ImageNet	No	33.3	87.9
WGAN (60000)	No	63.0	81.8
None	Yes	51.9	84.9
ImageNet	Yes	40.7	93.9
WGAN (60000)	Yes	66.7	93.9

**Table 4:** Classification result by difference in network models.

Model	Pretraining method	Classification accuracy [%]		
		Benign	Malignant	Overall
Proposed method (AlexNet)	None	51.9	84.9	70.0
	ImageNet	40.7	93.9	70.0
	WGAN (60000)	66.7	93.9	81.7

GoogLeNet	None	40.7	84.9	65.0
	ImageNet	48.2	87.9	70.0
	WGAN (60000)	48.2	97.0	75.0

VGG16	None	29.6	90.9	63.3
	ImageNet	33.3	87.9	63.3
	WGAN (60000)	48.2	84.9	68.3

## Data Availability

The source code and additional information used to support the findings of this study will be available from the corresponding author upon request.
